# Effects of valproic acid on skeletal metabolism in children with epilepsy: a systematic evaluation and meta-analysis based on 14 studies

**DOI:** 10.1186/s12887-020-1984-7

**Published:** 2020-03-02

**Authors:** Li Min, Wang Chunyan, Rong Biaoxue

**Affiliations:** 1 Nursing department, Binhe new district branch, Shenmu Hospital, Shenmu, Yulin City, Shaanxi China; 2 Department of Respiratory Medicine, Shenmu Hospital, Shenmu, Yulin City, Shaanxi China; 3 Department of Medicine, First Affiliated Hospital, Xi’an Medical University, Xi’an, China

**Keywords:** Valproic acid, Epilepsy; children, Bone metabolism, Bone mineral density, Meta-analysis

## Abstract

**Background:**

Previous studies have reported that long-term use of valproic acid can cause changes in bone metabolism in children. We conducted this meta-analysis to determine the effects of valproic acid on bone metabolism and bone mineral density (BMD) in children with epilepsy.

**Methods:**

Studies were searched from the databases of PubMed, Embase, Ovid, Cochrance Library, Springer Link and Web of Science. The effects of valproic acid on bone metabolism indicators and BMD were assessed through calculating the standardized mean difference (SMD) with 95% confidence interval (CI).

**Results:**

Fourteen studies with 987 individuals were included in this analysis. The long-term use of valproic acid did not affect the levels of serum calcium (*p* = 0.99), phosphorus (*p* = 0.28), ALP (*p* = 0.76), PTH (*p* = 0.36) and osteocalcin (*p* = 0.72), but it led to a decrease in 25-OH-VitD (*p* = 0.01) and BMD (*p* = 0.002 for the vertebra; *p* = 0.004 for the femur) in treating children with epilepsy.

**Conclusion:**

Long-term use of valproic acid in treating children with epilepsy can lead to a reduction in 25-OH-VitD and BMD. Measurements of 25-OH-VitD and BMD should be performed regularly in children taking the drug to detect early osteopenia caused by the drug.

## Background

As a chronic disorder of the brain, epilepsy affects people of all ages. Approximately 50 million people worldwide have epilepsy, making it one of the most common neurological diseases globally [1]. Epilepsy is common neurological disease in children. Although antiepileptic drugs (AEDs) do not have a “curative effect” on epilepsy, they control seizures. Valproic acid has been widely used as a long term anti epileptic medication [2], as it displays a certain effects in absence seizures, clinical depression, tonic-clonic seizures, juvenile myoclonic epilepsy and complex partial seizures [3, 4].

Height, weight, nutrition and cognitive status are closely related to children’s growth and development, especially bone metabolism. As early as 2004, it was reported that several factors affect bone metabolism in children with epilepsy, suggesting that long-term use of AEDs may cause damage to the skeletal system [5]. Cytochrome P450 (CYP450) isozyme can be induced by AEDs such as carbamazepine, phenytoin sodium, phenobarbital and valproic acid, which may lead to vitamin D deficiency, hypocalcemia, increased fracture risk [6]. Opinions suggest that long-term use of this drug may have an impact on bone metabolism in children [1–3, 7]. We performed a meta-analysis to determine the effects of valproic acid on bone metabolism and bone mineral density (BMD) in epileptic children.

## Methods

### Literature searching

Several databases including PubMed, Embase, Ovid, Cochrance Library, Springer Link and Web of Science were searched. The key words for searching literature included: “epilepsy,” “antiepileptic drugs,” “valproic acid,” “sodium valproate,” “bone metabolism,” “bone mineral density,” “bone density,” “children epilepsy,” and “AEDs”. The search strategy was to combine the topic of valproic acid with the topic of children epilepsy and to combine them with the indices of bone metabolism. We applied boolean operators, wildcards, and field identifiers to combine search terms. This meta-analysis was conducted based on the Reporting Items for Systematic Reviews and Meta-Analyses (PRISMA) statement [8].

### Inclusion criteria

The inclusion criteria used were: (1) The diagnosis of children with epilepsy which met diagnostic criteria of the International Anti-Epilepsy Alliance; (2) Children were under 18 years old and could participate in outdoor activities; (3) The design type must be cohort study or case-control study; (4) The valproic acid group must be treated with valproic acid monotherapy for more than 6 months; (5) The control group must be composed of healthy children not receiving any medications; and (6) The study must have at least one of the following study endpoints: serum calcium, phosphorus, alkaline phosphatase (ALP), parathyroid hormone (PTH), osteocalcin, and 25-hydroxy-vitamin D (25-OH-VitD) and bone mineral density (BMD).

### Exclusion criteria

The exclusion criteria used were: (1) Studies that contained children with encephalitis and other secondary epilepsy; (2) Lack of clear grouping **(**valproic acid group and healthy children group); (3) Children with epilepsy receiving other treatments; (4) Research costs were provided by drug manufacturers or studies initiated by drug producers; (5) The time of administration of valproic acid was not indicated, or the continuous medication was shorter than 6 months.

### Extraction of study variables

The extracted data included: (1) Author’s name, date of publication, and region of the research; (2) Study design and case number of different groups; (3) General demographic characteristics and baseline conditions; (4) The duration and average blood concentration of valproic acid; and (5) Bone metabolism indicators: calcium, serum phosphorus, ALP, PTH, osteocalcin, 25-OH-VitD and BMD.

### Methodological quality assessment

The quality of studies was evaluated using the Newcastle-Ottawa Scale (NOS) [9]. Each study was judged on eight items, categorized into three groups: the selection of the study groups; the comparability of the groups; and the ascertainment of either the exposure or outcome of interest for case-control or cohort studies respectively. A qualified project was awarded a star and the highest quality research will be awarded 9 stars [9, 10]. The two authors performed the evaluation independently. If there was disagreement, a third expert was invited to discuss and reach a consensus.

### Statistical analysis

The statistical methods involved in this study: (1) Chi-square test and I^2^ statistical test were used to assess the heterogeneity of included studies. If the *p*-value of the chi-square test was greater than 0.10, and the value of I^2^ was less than or equal to 50%, indicating low risk of heterogeneity, we choose the fixed-effect method of meta-analysis. Otherwise, we used a random effects model; (2) The standard mean difference (SMD) and 95% confidence interval (CI) were used to estimate the statistical effect; (3) The overall effect of the meta-analysis was tested using Z-scores with a significance of being set at *p* < 0.05; (4) Each study was removed from the estimated pool and analyzed to determine whether the addition and withdrawal of any study affected the overall statistical effect; (5) A funnel plot analysis, Begg’s test and Egger’s test were performed to evaluate the reliability of publication biases; (6) The SPSS (version 19.0, Chicago, USA) software was used to complete general statistics of measurement data; (7) Two computer software programs, Revman 5.2 (Cochrane collaboration) and Stata 14.0 (Stata Corporation, TX, USA), were used to perform the meta analysis of measurement data.

## Results

### A total of 14 studies were included in this meta-analysis

**Fig. 1:**
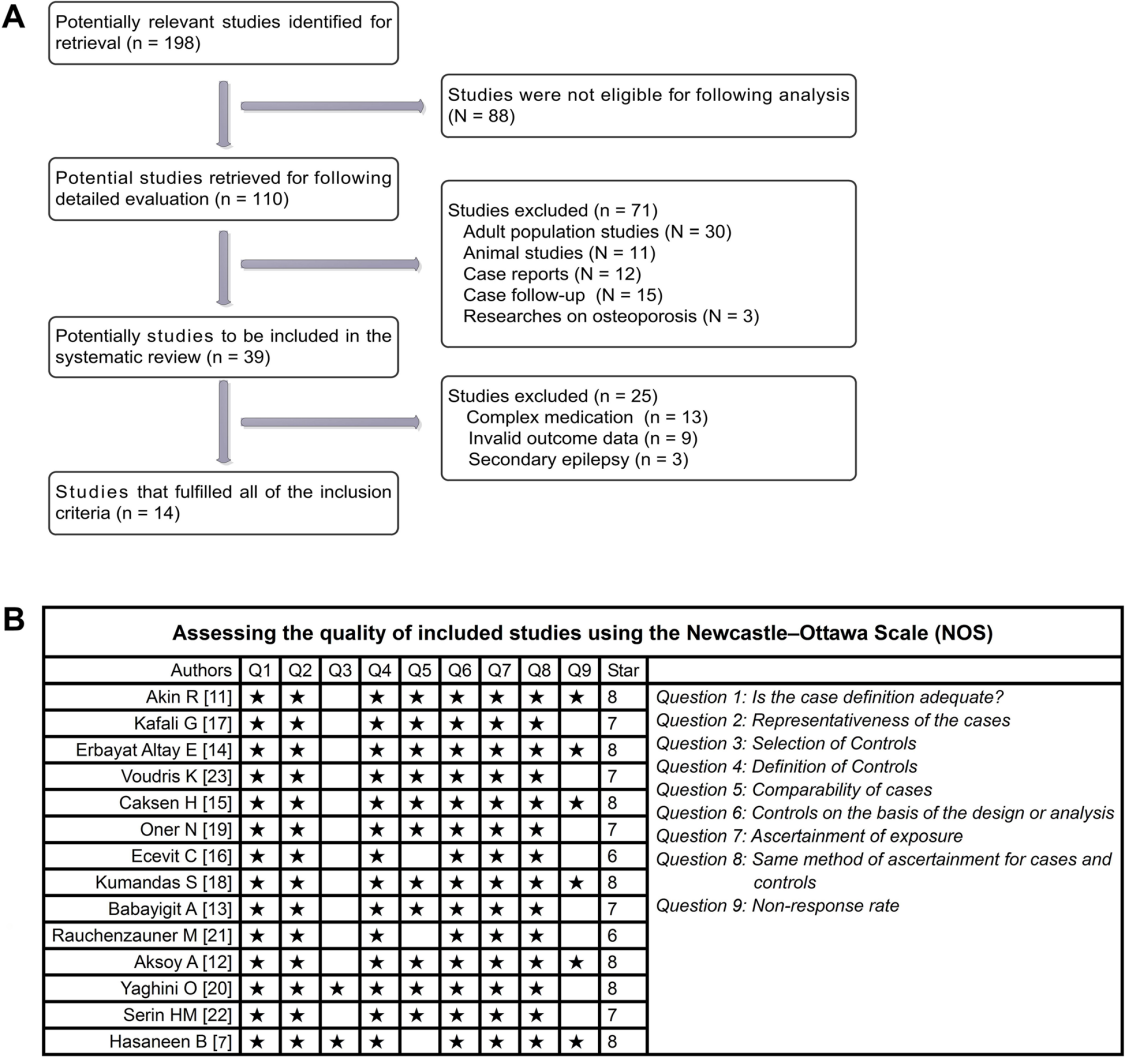
Flow chart of selection process and quality assessment. **a** After screening, 14 studies met the inclusion criteria. **b** The NOS Scale showed that these studies had a moderate to high quality. NOS, Newcastle-Ottawa Scale

Of the initial 198 studies, 88 did not meet inclusion criteria (reviews, summary of meetings and non-human studies). Of the remaining 110, 71 were excluded (adult population studies, animal research, case reports, lost to follow-up and reports on osteoporosis). Of the remaining 39, 25 were excluded (complicated treatment, invalid outcome and research on secondary epilepsy) (Fig. 1a). Finally, a total of 14 [11–23] were recruited in this meta-analysis (Table 1).

**Table 1:** Description of the included studies

Authors	Year	Cases (N)			Age (Years)		Gender (Male/female) (N)				Weight (kg)		Height (cm)	
		All	Valproic acid	control	Valproic acid	Control	Valproic acid		Control		Valproic acid	Control	Valproic acid	Control
Akin R [11]	1998	51	25	26	8.8 ± 0.5	8.9 ± 0.6	14	11	15	11	31.9 ± 2.06	29 ± 1.8	132 ± 13.4	132 ± 3.4
Kafali G [17]	1999	76	19	57	6–12	6–12	10	9	29	28	28.1 ± 8.3	27.5 ± 6.5	130 ± 10	130 ± 10
Erbayat Altay E [14]	2000	37	15	22	5.5–18	7–15.5	5	10	8	14	15.5 (34.67)	20–74(37.43)	95–69(135)	112–170(142.73)
Voudris K [23]	2002	94	47	47	8.1 ± 3.9	Comparable	–	–	–	–	–	–	–	–
Caksen H [15]	2002	53	31	22	9.0 ± 3.1	9.8 ± 4.2	17	14	12	10	–	–	–	–
Oner N [19]	2004	66	33	33	7.1 ± 3.5	7.4 ± 2.8	–	–	–	–	–	–	121 ± 22.2	123 ± 17.3
Ecevit C [16]	2004	47	16	31	10.6 ± 3.2	11.5 ± 2.6	–	–	–	–	34.56 ± 8.42	40.91 ± 12.44	138 ± 12	146 ± 15
Kumandas S [18]	2006	55	33	22	8.8 ± 2.0	8.9 ± 2.3	17	16	20	13	32.8 ± 11.9	31.5 ± 10.5	132.2 ± 10.7	123 ± 28.4
Babayigit A [13]	2006	61	31	30	11.2 ± 4.1	13.1 ± 3.1	15	16	14	16	38.15 ± 16.89	47.98 ± 15.49	143.6 ± 22	155.7 ± 19.14
Rauchenzauner M [21]	2010	126	85	41	12.4 ± 3.3	12.1 ± 3.5	38	47	28	12	–	–	–	–
Aksoy A [12]	2011	103	53	50	8.4 ± 2.0	8.2 ± 1.9	25	28	31	19	27.85 ± 8.49	27.49 ± 6.42	125 ± 0.12	127 ± 0.10
Yaghini O [20]	2014	60	30	30	7.4 ± 2.4	7.9 ± 2.4	–	–	–	–	–	–	–	–
Serin HM [22]	2015	48	28	20	8.11 ± 3.95	7.6 ± 3.3	11	17	13	7	–	–	–	–
Hasaneen B [7]	2017	101	21	80	6.2 ± 3.1	8.7 ± 3.4	13	8	41	39	–	–	–	–

N, cases

### Included studies show good homogeneity and comparability

Fourteen studies [11–23] published between 1998 and 2017 with a total 978 children were included in this meta-analysis (Table 1). Studies ranged in size from 37 [14] - 126 cases [21] with 467 in the valproate group and 511 cases in the control group. Boys accounted for 48.4% in the valproic acid group and 55.5% in control group (*p* > 0.05). Seven studies showed data on height and weight [11–14, 16–18] with no statistical differences between the valproic acid group and the control group (*p* > 0.05).

### Included studies show moderate to high research quality

Fourteen studies [11–23] were retrospective single-center studies. Ten studies [11–19, 22] originated from Turkey, and the other four studies from Greece [23], Austria [21], Iran [20] and Egypt [7] (Table 2). Six studies [7, 11, 12, 14, 15, 18] provided high quality research data. Seven studies [11, 12, 15, 16, 18, 19, 23] reported serum level of valproic acid. Eleven studies [11–15, 17–20, 22, 23] showed a low risk of selection bias. In summary, seven studies [7, 11, 12, 14, 15, 18, 20] received eight stars, five [13, 17, 19, 22, 23] received seven and two [16, 21] were six (Fig. 1b).

**Table 2:** Methodology and quality of inclined studies

Authors	Year	Research design	Country	Treatment duration /years	Serum drug level (mg/mL)	Bone metabolism index
Akin R [11]	1998	Retrospective	Turkey	2.4 ± 0.2	66 ± 2.2	Ca, P, ALP, BMD
Kafali G [17]	1999	Retrospective	Turkey	> 0.5	–	ALP, BMD
Erbayat Altay E [14]	2000	Retrospective	Turkey	3.1 + 1.8 (1–6)	–	BMD
Voudris K [23]	2002	Retrospective	Greece	> 0.5	59.1 ± 2.7	ALP
Caksen H [15]	2002	Retrospective	Turkey	1.93 ± 1.90	85.29 ± 11.72	Ca, P, ALP, PTH, OC
Oner N [19]	2004	Retrospective	Turkey	> 0.5	56.8 ± 15.5	Ca, P, BMD
Ecevit C [16]	2004	Retrospective	Turkey	> 0.5	53.75 ± 23.94	BMD
Kumandas S [18]	2006	Retrospective	Turkey	2.9 ± 1.2	67.37 ± 8.31	Ca, P, ALP, BMD, PTH, OC, 25-OH-VitD
Babayigit A [13]	2006	Retrospective	Turkey	> 1	–	Ca, P, ALP, BMD, PTH, 25-OH-VitD
Rauchenzauner M [21]	2010	Retrospective	Austria	> 0.5	–	Ca, P, 25-OH-VitD
Aksoy A [12]	2011	Retrospective	Turkey	3.23 ± 1.09	56.96 ± 7.34	Ca, P, ALP, BMD, OC, 25-OH-VitD
Yaghini O [20]	2014	Retrospective	Iran	> 0.5	–	Ca, P, ALP, BMD, PTH
Serin HM [22]	2015	Retrospective	Turkey	> 0.5	–	Ca, P, ALP, BMD, PTH
Hasaneen B [7]	2017	Retrospective	Egypt	1.3 ± 0.6	–	Ca, P, BMD, PTH

*Ca* Serum calcium, *P* Serum phosphorus, *ALP* Alkaline phosphatase, *BMD* Bone mineral density, *PTH* Parathyroid hormone, *OC* Osteocalcin, *25-OH-VitD* 25-hydroxy-vitamin D

### Valproic acid does not affect the levels of serum calcium and phosphorus in children with epilepsy

**Fig. 2:**
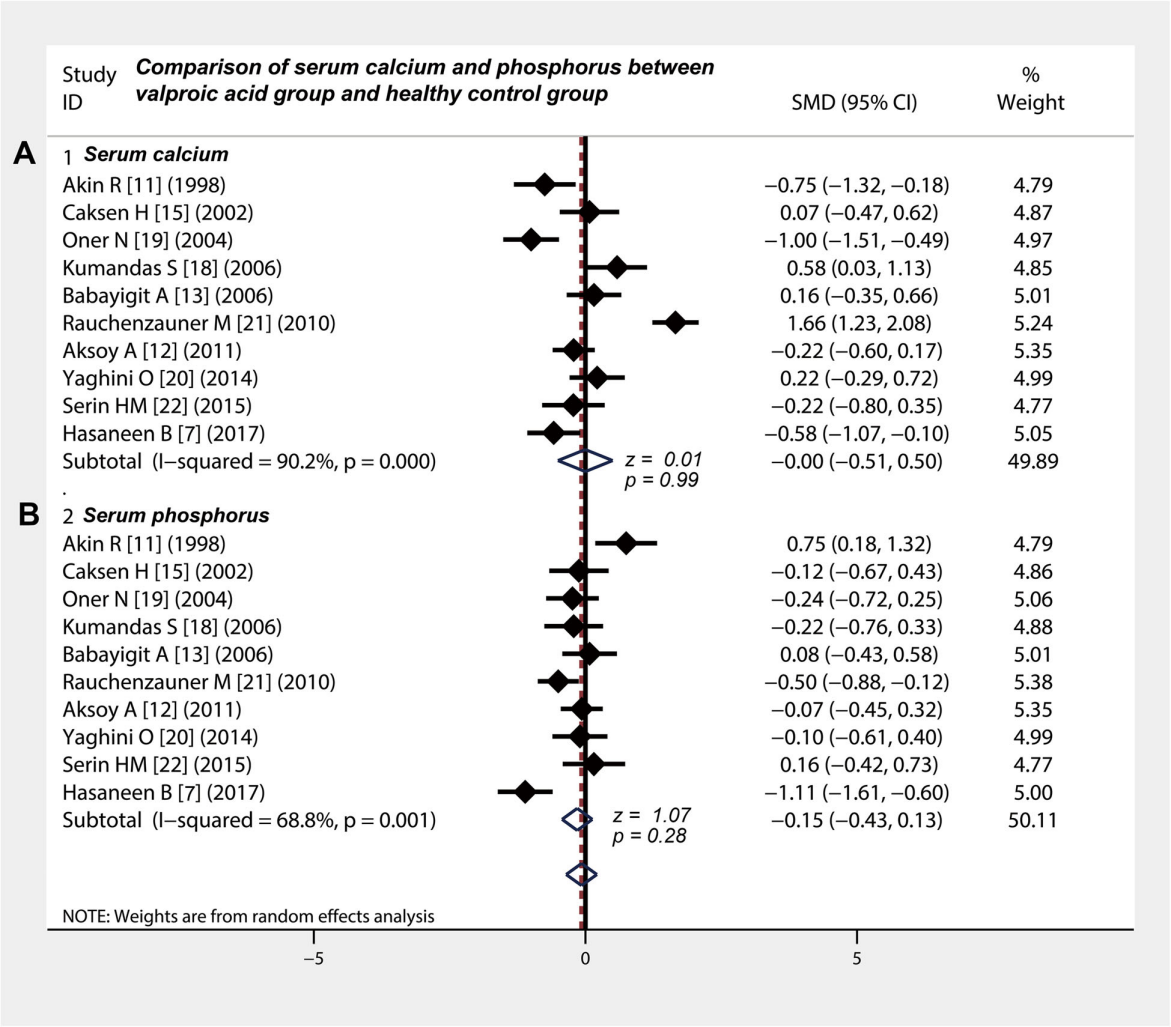
Comparison of serum calcium and phosphorus between valproic acid group and healthy control group. **a** and **b** There was no significant difference in serum calcium and phosphorus between valproic acid group and control group. CI, confidence interval

Ten studies [7, 11–13, 15, 18–22] compared the level of serum calcium between valproic acid group and healthy control group and showed a chi-square value of 91.77 (degrees of freedom = 9; *p* < 0.001) and *I*-square value of 90.2% (Table 3). The weight of included studies ranged from 4.77 to 5.35%, and the pooled SMD was − 0.00 (95% CI: − 0.51 to − 0.50) (Fig. 2a), showing that valproic acid did not affect the level of serum calcium in children with epilepsy (z = 0.01, *P* = 0.99). Ten studies [7, 11–13, 15, 18–22] compared the serum phosphorus level and showed a chi-square statistic of 28.89 (degrees of freedom = 9; *p* = 0.001) and *I*-square of 68.8% (Table 3). The combined SMD was − 0.15 (95% CI: − 0.43 to 0.13), suggesting that valproic acid did not affect the level of serum phosphorus in children with epilepsy (z = 1.07, *P* = 0.28) (Fig. 2b).

**Table 3:** The effect of valproic acid on bone metabolism in epileptic children

Author	Bone metabolism index (Mean ± standard deviation)											
	Serum calcium (mg/dL)		Serum phosphorus (mg/dL)		Alkaline phosphatase (U/L)		Parathyroid hormone (pg/mL)		Osteocalcin (Inconsistent)^d^		25-hydroxy-vitamin D (ng/ml)	
	Valproic acid	Control	Valproic acid	Control	Valproic acid	Control	Valproic acid	Control	Valproic acid	Control	Valproic acid	Control
Akin R [11]	9.08 ± 0.1	9.42 ± 0.1	5.39 ± 0.2	5.24 ± 0.2	194 ± 18^c^	204 ± 11^c^	–	–	–	–	–	–
Kafali G [17]	–	–	–	–	569.35 ± 263.35	303.7 ± 109.8	–	–	–		–	–
Erbayat Altay E [14]	–	–	–	–	–	–	–	–	–	–	–	–
Voudris K [23]	–	–	–	–	162.3 ± 71.8	144.9 ± 53.3	–	–	–	–	–	–
Caksen H [15]	9.84 ± 0.62	9.80 ± 0.39	5.02 ± 0.72	5.10 ± 0.58	481.51 ± 165.56	554.77 ± 210.41	35.38 ± 31.06	33.14 ± 17.18	3.13 ± 3.22	3.11 ± 2.43	–	–
Oner N [19]	10.02 ± 0.4^a^	10.42 ± 0.4^a^	4.87 ± 0.8	5.1 ± 1.1	–	–	–	–	276.3 ± 136.6	201.4 ± 82.9	–	–
Kumandas S [18]	9.9 ± 0.3	9.7 ± 0.4	4.4 ± 0.5	4.5 ± 0.4	561 ± 166	487 ± 82	39.1 ± 12.8	36.3 ± 4.9	–	–	15.1 ± 3.5^e^	16.6 ± 4.7^e^
Babayigit A [13]	9.95 ± 0.41	9.89 ± 0.35	3.53 ± 0.57	3.49 ± 0.47	506.3 ± 215.1	436.5 ± 147.9	43.46 ± 22.1	38.17 ± 14.8	–	–	17.87 ± 7.27	22.3 ± 7.1
Rauchenzauner M [21]	9.89 ± 0.32^a^	9.37 ± 0.3^a^	4.2 ± 0.6^b^	4.5 ± 0.6^b^	–	–	–	–	–	–	43.2 ± 25.6	49.9 ± 33.8
Aksoy A [12]	9.84 ± 0.38	9.93 ± 0.45	4.87 ± 0.44	4.90 ± 0.44	422.74 ± 177.52	518.34 ± 158.82	–	–	100.27 ± 34.09	120.93 ± 41.57	37.68 ± 30.73	38.82 ± 27.33
Yaghini O [20]	9.67 ± 0.37	9.58 ± 0.46	4.78 ± 0.75	4.84 ± 0.36	204.10 ± 58	270.13 ± 106	54.82 ± 16.33	80.09 ± 49.35	–	–	–	–
Serin HM [22]	9.42 ± 0.44	9.5 ± 0.2	4.9 ± 0.5	4.8 ± 0.8	202.36 ± 65.87	183.7 ± 50.9	44.29 ± 20.04	35.1 ± 13	–	–	–	–
Hasaneen B [7]	7.9 ± 1.2	8.6 ± 1.2	4.5 ± 0.8	5.3 ± 0.7	–	–	38.1 ± 27.2	29.6 ± 7.4	–	–	–	–

^a^Data has been converted, Ca^2+^: mmol/L × 4.008 = mg/dL; ^b^Data has been converted, P: mmol/L × 3.097 = mg/dL; ^c^Data has been converted, mU/ml ×  1000/1000 = U/L; ^d^Different detection methods cannot be used to achieve unity; ^e^Data has been converted, ug/L× 1000/1000 = ng/ml

### Levels of serum ALP and PTH in children with epilepsy are not affected by the long-term use of valproic acid

**Fig. 3:**
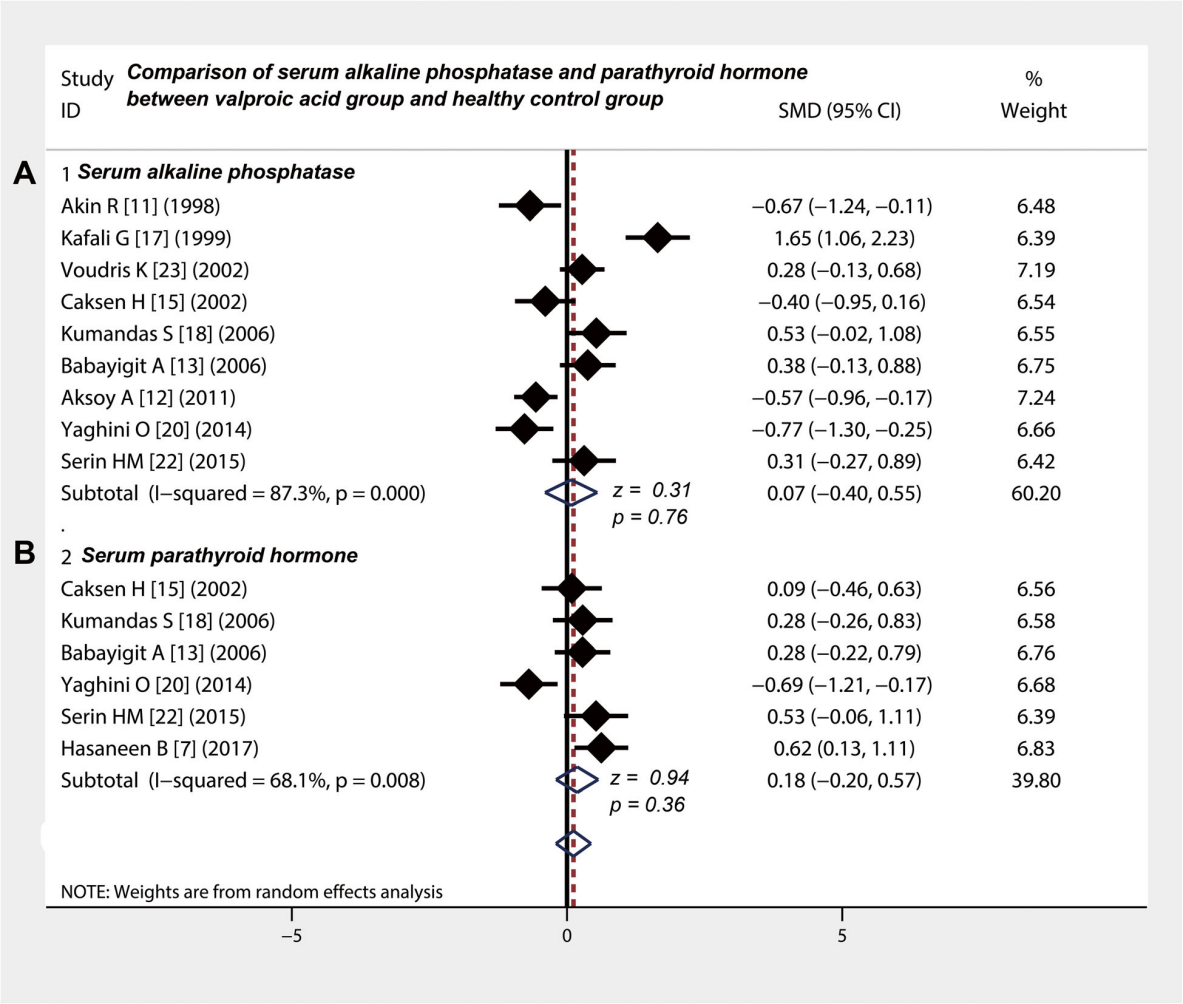
Comparison of serum alkaline phosphatase and parathyroid hormone between valproic acid group and healthy control group. **a** and **b** Valproic acid did not affect the levels of serum alkaline phosphatase and parathyroid hormone in children with epilepsy. CI, confidence interval

To compare the levels of serum ALP and PTH, the chi-square values were 62.9 and 15.69 (*p* = 0.008 and *p* < 0.001) and the *I*-square values were 87.3 and 68.1%. Nine studies [11–13, 15, 17, 18, 20, 22, 23] compared the level of serum ALP between valproic acid group and control group (Table 3). The combined SMD was 0.07 and the 95% CI was − 0.40 to 0.55, showing that valproic acid did not affect the level of serum ALP in children with epilepsy (z = 0.31, *P* = 0.76) (Fig. 3a). Six studies [7, 13, 15, 18, 20, 22] compared the serum level of PTH (Table 3) and the combined SMD was 0.18 (95% CI: − 0.20 to 0.43; z = 0.94, *P* = 0.36), showing that valproic acid did not affect the level of serum PTH in children with epilepsy (Fig. 3b).

### Level of serum osteocalcin in children with epilepsy is not affected by the long-term use of valproic acid

**Fig. 4:**
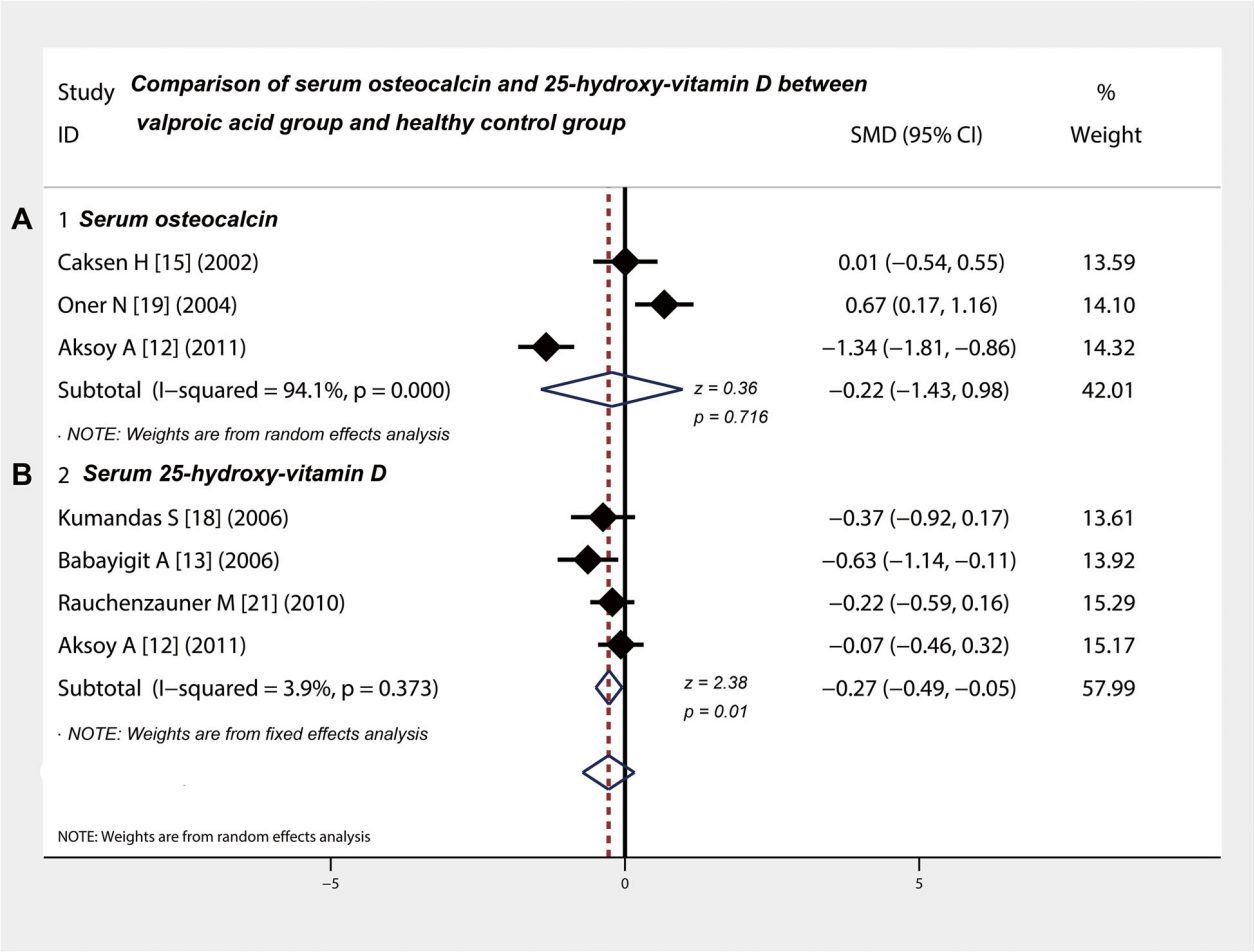
Comparison of serum osteocalcin and 25-hydroxy-vitamin D between valproic acid group and healthy control group. **a** and **b** Osteocalcin and 25-hydroxy-vitamin D in children with epilepsy were not affected by the long-term use of valproic acid. CI, confidence interval

To compare the levels of serum osteocalcin, the chi-square value was 43.21 (*p* < 0.001) and the *I*-square values was 94.1%. Three studies [12, 15, 19] compared the serum osteocalcin level between valproic acid group and control group (Table 3). The combined SMD was − 0.22 (95% CI: − 1.43 to 0.98; z = 0.36, *P* = 0.72), which suggested that the level of serum osteocalcin in children with epilepsy was not affected by the long-term use of valproic acid (Fig. 4a).

### Long-term use of valproic acid leads to a decline of serum 25-OH-VitD level in epilepsy children

To compare the level of 25-OH-VitD, the chi-square value was 3.12 (*p* = 0.373) and the *I*-square value of 3.9%. Four studies [12, 13, 18, 21] compared the level of 25-OH-VitD between in the valproic acid group and healthy control group (Table 3). The pooled SMD was − 0.27 (95% CI: − 0.49 to − 0.05) (Fig. 4b), suggesting that the level of 25-OH-VitD in valproic acid group was significantly lower than that in control group (z = 2.38, *P* = 0.01).

### Long-term use of valproic acid reduces the BMD in children with epilepsy

**Fig. 5:**
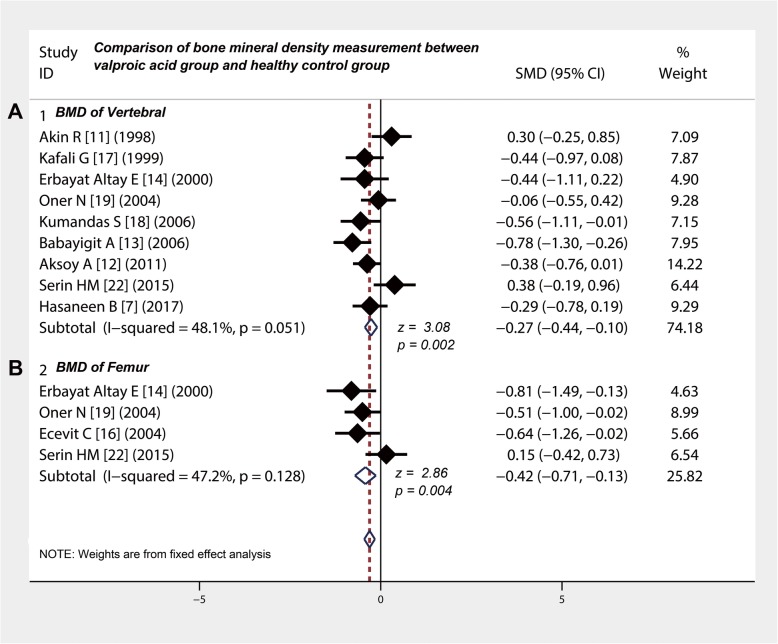
Comparison of bone mineral density between valproic acid group and healthy control group. **a** and **b** The BMD of vertebral and femur in valproic acid group was significantly lower than that in the control group. CI, confidence interval; BMD, bone mineral density

The heterogeneity test on BMD of lumbar vertebrae and femur in 9 studies [7, 11–14, 17–19, 22] did not show statistical significance (*p* = 0.051 and 0.018). The BMD of lumbar vertebrae in valproic acid group and control group were measured in 9 studies [7, 11–14, 17–19, 22] (Table 4) and showed that the BMD of vertebral in valproic acid group was significantly lower than that in the control group (SMD = − 0.27, 95% CI: − 0. 44 to − 0.10, *P* = 0. 002) (Fig. 5a). Four studies [14, 16, 19, 22] compared the BMD of the femur (Table 4). The pooled SMD was − 0.42 (95% CI: − 0.71 to − 0.13) (Fig. 5b), indicating that the BMD of the femur in valproic acid group was significantly lower than that in control group (z = 2.86, *P* = 0.004).

**Table 4:** The effect of valproic acid on bone mineral density in epileptic children

Author	Year	Testing method	Testing Equipment	Bone mineral density (Mean ± standard deviation)					
				Cases (N)		Vertebral (gm/cm^2^)		Femur (gm/cm^2^)	
				Valproic acid	Control	Valproic acid	Control	Valproic acid	Control
Akin R [11]	1998	DXA	DXA Norland (Fort Atkinson, WI) XR-36 densitometer	25	26	0.574 ± 0.02	0.568 ± 0.02	–	–
Kafali G [17]	1999	DXA	DEXA, QDR 4500 W Acclaim,Hologic	19	57	0.517 ± 0.07	0.548 ± 0.07	–	–
Erbayat Altay E [14]	2000	DXA	DXA Norland (Fort Atkinson, WI) XR-36 densitometer	15	22	0.596 ± 0.09	0.624 ± 0.08	0.66 ± 0.08	0.72 ± 0.07
Oner N [19]	2004	DEXA	Norland XR-36 analyzer, Norland Medical System, USA	33	33	0.56 ± 0.2	0.57 ± 0.1	0.61 ± 0.2	0.69 ± 0.1
Ecevit C [16]	2004	DXA	XR-36, Norland, Hologic (Medical Equipment Company, Minster, OH)	16	31	–	–	0.67 ± 0.08	0.73 ± 0.1
Kumandas S [18]	2006	DEXA	Hologic QDR 4500 Elite DEXA (USA)	33	22	0.60 ± 0.09	0.65 ± 0.09	–	–
Babayigit A [13]	2006	DXA	Hologic QDR 4500 W bone densitometer	31	30	0.665 ± 0.20	0.807 ± 0.13	–	–
Aksoy A [12]	2011	DXA	QDR-2000/W densitometer (Hologic, Waltham, MA, USA)	53	50	0.58 ± 0.08	0.61 ± 0.08	–	–
Serin HM [22]	2015	DXA	Discovery Xi, Hologic	28	20	0.58 ± 0.13	0.53 ± 0.13	0.6 ± 0.13	0.58 ± 0.13
Hasaneen B [7]	2017	Dual photon X-ray absorptiometry	DXA; Lunar DPX IQ-USA	21	80	0.63 ± 0.17	0.68 ± 0.17	–	–

*DXA* Dual-energy x-ray absorptiometry, *DEXA* Dual-energy X-ray absorptiometry

### Analysis of sensitivity and publication bias

**Fig. 6:**
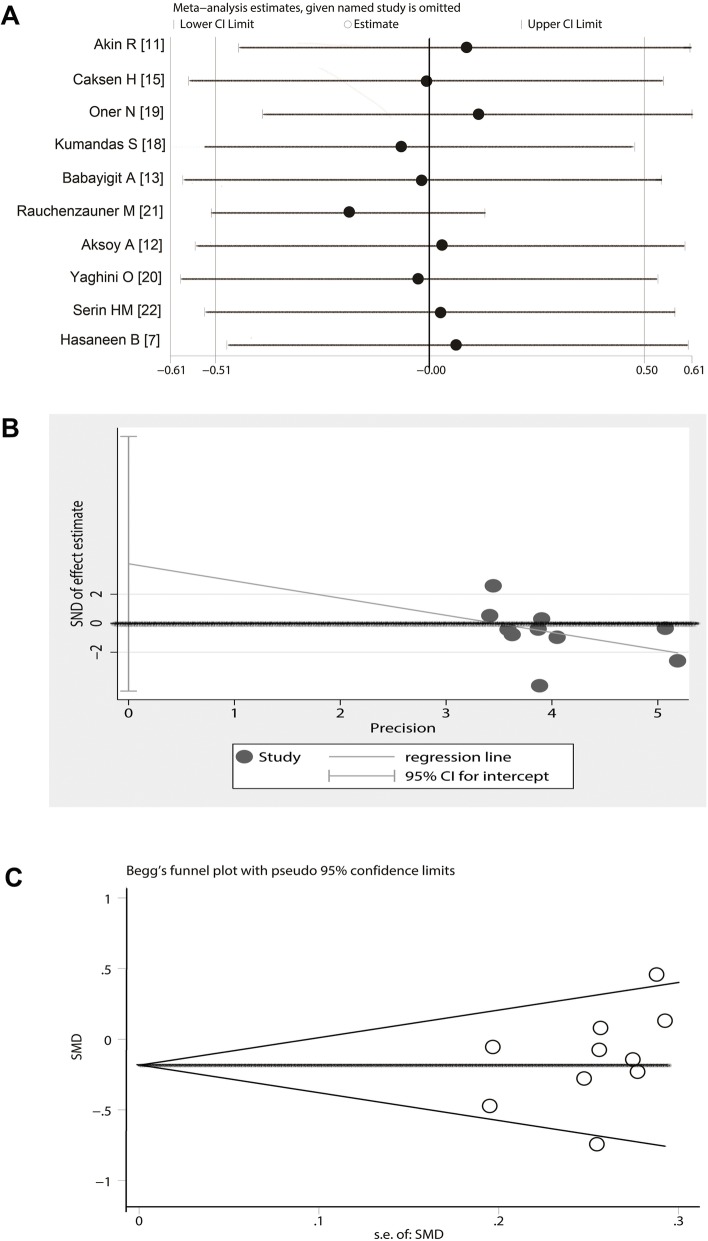
Analysis of sensitivity and publication bias on included studies. **a** Sensitivity analyses suggested that the exclusion of any study did not substantially alter the estimates and the final statistical performance. **b** The Egger’s test showed that the discrete graph was basically distributed on both sides of the estimated line. **c** The funnel plot produced by begg’s test showed that the included studies were symmetrically distributed on both sides of the bottom of the funnel plot

Sensitivity analysis suggested that the exclusion of any study did not substantially alter the estimates and affect the final statistical analysis. The SMD pool oscillated between − 1.11 and 0.75 (Fig. 6a). The Egger’s test showed that the *T* value was − 1.26 (Pr > |t| = 0.313) and the Begg’s Test showed that the Std. Dev. of Score was 11.18 (Pr > |z| = 0.152). The discrete graph produced by Egger’s test indicated that included studies were basically distributed on both sides of the estimated line (Fig. 6b). The funnel plot produced by Begg’s Test showed that the included studies were symmetrically distributed on both sides of the bottom of the funnel plot (Fig. 6c). Based on the above evidence, there is a very small probability of publication bias in these studies.

## Discussion

In addition to the influence of nutritional status, the metabolism of the skeletal system in children is also affected by multiple factors such as chronic disease, drugs, and outdoor exercise time [24, 25]. Valproic acid has been used as one of the traditional agents to control epileptic seizures in children [2, 13]. As early as 2003, one study proposed that the long-term use of AEDs may cause damage to the skeletal system [19], and was followed by several multi disciplinary research studies on this topic [7, 12–15, 18–20, 23]. As a liver enzyme inhibitor, valproic acid has been reported to affect bone metabolism, but results are inconsistent [11–23]. We collected observational studies on children with epilepsy receiving valproic acid to assess the potential effects of this medication on bone metabolism.

A total of 14 studies [11–23] were included in our analysis. We found that the long-term use of valproic acid did not affect the levels of blood calcium, phosphorus, ALP, PTH and osteocalcin in epileptic children [26]. Our study showed that the level of 25-OH-VitD in valproic acid group was significantly lower than that of the healthy control group, which implies that valproic acid may affect bone metabolism by downregulating the level of 25-OH-VitD. Through the action of liver 25-hydroxylase, human exogenous and endogenous Vitmin D2 and D3 are converted into 25-OH-VitD, which requires the action of 1-α hydroxylase in the proximal tubule of the kidney to form active 1–25-OH-VitD [27]. Previous studies suggest that both valproic acid and its intermediate metabolite 4-ene-VPA can be hepatotoxic, especially to mitochondrial function, which can affect the hydroxylation function of liver and kidney through inhibiting the activity of P450 isozyme CYP2C191 and uridine diphosphate transferase, thus further affect the synthesis of 1, 25-OH-VitD [28, 29].

Dual-energy x-ray absorptiometry (DEXA) has been recommended to measure the BMD of children [30]. Fourteen studies included in our meta-analysis all used DEXA as a measure of BMD, and results indicated that there was very good homogeneity and comparability between these studies. We found that for both the vertebrae and femur, the BMD of the valproic acid group was significantly lower than that in the healthy control group, which implies that long-term use of valproic acid decreased the BMD of children with epilepsy. Testing the BMD could help find changes in bone structure in the early stages in children with epilepsy who taken valproic acid, thus providing the clue for early treatment [31]. Long-term use of AEDs may decrease the level of vitamin D, thus reduce the BMD and affect bone structure. Early administration of calcium and vitamin D could prevent bone damage [32].

The 14 studies included in this meta-analysis were case-control studies with good homogeneity [11–23]. A NOS assessment showed that 12 studies had obtained more than seven stars. Publication bias analysis showed that these studies were unlikely to have publication bias. However, these studies have some shortcomings: first, the samples in most studies were relatively small; second, the methods and units of measurement used for bone metabolism indicators differed in these studies, which could lead to methodological heterogeneity; third, the relatively large number of studies from turkey, which may produce geographical bias; fourth, most studies did not focus on the correlation between the plasma concentration of valproic acid and bone metabolism indicators, nor did they compare the correlation between different medication time and bone metabolism indicators.

## Conclusion

The long-term use of valproic acid has no significant effect on serum calcium, phosphorus, ALP, PTH, and osteocalcin in children with epilepsy, but it can cause a decrease of 25-OH-VitD and BMD, suggesting that children with epilepsy who had taken valproic acid for a long time have a risk of osteopenia, but multiple centers and large sample data are still needed to look for specific indicators of early bone damage.

## Data Availability

The datasets supporting the conclusions of this article are included within the article.

## Abbreviations

25-OH-VitD: 25-hydroxy-vitamin D
AEDs: Antiepileptic drugs
ALP: Alkaline phosphatase
BMD: Bone mineral density
CI: Confidence interval
CYP450: Cytochrome P450
DEXA: Dual-energy x-ray absorptiometry
M ± SD: Mean ± standard deviation
NOS: Newcastle-Ottawa Scale
PTH: Parathyroid hormone
SMD: Standardized mean difference
